# How should we be using biomarkers in trials of disease modification in Parkinson’s disease?

**DOI:** 10.1093/brain/awad265

**Published:** 2023-08-03

**Authors:** Nirosen Vijiaratnam, Thomas Foltynie

**Affiliations:** Department of Clinical and Movement Neurosciences, UCL Queen Square Institute of Neurology, London WC1N 3BG, UK; Department of Clinical and Movement Neurosciences, UCL Queen Square Institute of Neurology, London WC1N 3BG, UK

**Keywords:** Parkinson’s disease, biomarkers, disease modification, clinical trials

## Abstract

The recent validation of the α-synuclein seed amplification assay as a biomarker with high sensitivity and specificity for the diagnosis of Parkinson’s disease has formed the backbone for a proposed staging system for incorporation in Parkinson’s disease clinical studies and trials. The routine use of this biomarker should greatly aid in the accuracy of diagnosis during recruitment of Parkinson’s disease patients into trials (as distinct from patients with non-Parkinson’s disease parkinsonism or non-Parkinson’s disease tremors). There remain, however, further challenges in the pursuit of biomarkers for clinical trials of disease modifying agents in Parkinson’s disease, namely: optimizing the distinction between different α-synucleinopathies; the selection of subgroups most likely to benefit from a candidate disease modifying agent; a sensitive means of confirming target engagement; and the early prediction of longer-term clinical benefit. For example, levels of CSF proteins such as the lysosomal enzyme β-glucocerebrosidase may assist in prognostication or allow enrichment of appropriate patients into disease modifying trials of agents with this enzyme as the target; the presence of coexisting Alzheimer’s disease-like pathology (detectable through CSF levels of amyloid-β_42_ and tau) can predict subsequent cognitive decline; imaging techniques such as free-water or neuromelanin MRI may objectively track decline in Parkinson’s disease even in its later stages. The exploitation of additional biomarkers to the α-synuclein seed amplification assay will, therefore, greatly add to our ability to plan trials and assess the disease modifying properties of interventions. The choice of which biomarker(s) to use in the context of disease modifying clinical trials will depend on the intervention, the stage (at risk, premotor, motor, complex) of the population recruited and the aims of the trial. The progress already made lends hope that panels of fluid biomarkers in tandem with structural or functional imaging may provide sensitive and objective methods of confirming that an intervention is modifying a key pathophysiological process of Parkinson’s disease. However, correlation with clinical progression does not necessarily equate to causation, and the ongoing validation of quantitative biomarkers will depend on insightful clinical-genetic-pathophysiological comparisons incorporating longitudinal biomarker changes from those at genetic risk with evidence of onset of the pathophysiology and those at each stage of manifest clinical Parkinson’s disease.

## Teaser

Vijiaratnam and Foltynie consider how fluid and imaging biomarkers can best be used in Parkinson’s disease trials to help select participants most likely to benefit from candidate disease-modifying agents, confirm target engagement, and track disease progression and treatment response.

## Introduction

Modifying the relentless deteriorating course of Parkinson’s disease (PD) remains a critical yet currently elusive goal. Despite decades of trials evaluating promising candidates, no treatments have yet been proven to achieve this. While this may be due to lack of trial evaluation of truly effective agents, other potentially contributing factors include imprecise patient selection, inadequacies in trial design, failure to confirm target engagement and the absence of objective measures of disease progression.^1^

One way of improving the likelihood of success is by identifying better biomarkers. A biomarker is a characteristic that is objectively measured and evaluated from any substance, structure or process that can be measured in the body or its products as an indicator of normal biological or pathogenic processes, or pharmacologic responses to a therapeutic intervention.^2^ An ideal biomarker should be readily quantifiable in accessible clinical samples [clinical assessments, biofluids (blood, CSF, urine, saliva, tears, stool), imaging and tissues (skin, oro-gastrointestinal mucosa)], while being reliable, quick and inexpensive.

Suboptimal patient selection in disease modifying trials may be related to poor diagnostic accuracy. Pathological modification (phosphorylation and conformational transformation) of the physiological protein α-synuclein (α-syn) to misfolded oligomeric and fibrillary forms is the most consistent pathological feature of PD.^3^ The accumulation and interplay of these abnormal protein forms with the organelles/cellular pathways involved in their clearance as well as normal cellular maintenance and survival results in neuronal dysfunction and ultimately axonal injury and neuronal death.

The α-syn seed amplification assay (SAA) has high sensitivity and specificity for PD diagnostic accuracy, with a recent study of >1100 samples from the Parkinson’s progression markers initiative (PPMI) cohort^4^ further confirming pre-existing evidence for its use,^5–11^ and is now proposed as a core aspect of a potential staging system for PD.^12,13^ This is potentially a pivotal step in clarifying eligibility criteria for inclusion in trials and distinguishing PD patients from those with atypical forms of parkinsonism. While needing further clarification, the α-syn SAA is at the present time largely a binary measure simply indicating the presence/absence of the pathophysiological process of α-syn aggregation and cannot yet be used to track disease severity, which instead relies on clinical measurements.

As such, there is still a need for additional biomarkers that might enrich treatment arms for PD subgroups most likely to respond and allow early exploratory analyses according to engagement of the therapeutic with its putative target. Current trials typically rely on clinical end points with scales and questionnaires, which are subject to inter-rater variability, while potentially being confounded by symptomatic drug effects. Evaluations using scales may also be compromised by non-linear changes over time,^14^ be limited by reduced compliance, recall bias and fatigue,^15^ sometimes do not correlate sufficiently with quantitative objective assessments^16,17^ and vary in their sensitivity at different disease stages,^18,19^ raising questions about the inclusion of patients who may have progressed beyond the salvageable period.

Biomarkers that are robustly demonstrated to track disease progression and treatment effects could potentially shorten periods of assessment and reduce the number of patients required for preliminary demonstration of efficacy. Ideally, short-term changes in the biomarker should anticipate long-term clinical outcomes. Furthermore, by confirming target engagement according to the dose(s) of the agent under study, biomarkers can be used to improve the distinction between an intervention’s disease-modifying effects and purely symptomatic improvements. While there are parallel efforts exploring additional biomarkers for PD prior to clinically manifest disease, in this review, we will discuss the current state of fluid, tissue and imaging biomarker developments in clinically established PD and their potential for use either alone or in combination in future disease modifying clinical trials.

## Fluid and tissue biomarkers

Box 1 outlines techniques that have been used to measure different forms of α-syn as well as other protein/enzyme levels that reflect cellular pathway abnormalities that can be measured in biofluids.

Box 1 **Fluid and tissue biomarker measurement techniques**

**ELISA**

- target-specific antibodies bind to the sample proteins
- secondary antibody linked to an enzyme recognizes the matched antibodies
- fluorescent reaction is created when exposed to a chemical substrate
- amount of antigen present correlates to intensity of colour change
- detection range inferior to other high-sensitivity techniques

**Luminex**

- beads conjugated with antibody against specific analyte present different colour codes
- high-throughput screening
- can measure up to 80 different proteins or RNA from a single microplate

**Mesoscale discovery**

- high-throughput measurement of single or multiple targets
- antibodies can be conjugated to generate electro chemiluminescent signals, unlike ELISA

**Single molecule array**

- antibody-based ELISA and bead-based platform
- antibody-coated bead binds to a single molecule and analysed separately
- multiplexing of up to 11 analytes, high sensitivity and wide detection range

**Proximity extension assay**

- DNA oligonucleotide tags linked to matched antibodies that both bind to target protein
- antibodies come into proximity on binding, DNA duplex formed, sequence amplified
- wide library of matched antibodies with high sensitivity and specificity for their targets

**SomaScan**

- Aptamers (short, single-stranded DNA or RNA molecules) bind target
- quantified by microarrays or quantitative PCR
- allows creation of library with high sensitivity for targets

**Single molecule counting**

- antibody–antigen sandwich complexes from either beads or plates
- broken up and fluorescently labelled detection antibody counted by laser beam
- allows for a high dynamic concentration range

**Mass spectrometry**

- measures mass-to-charge ratio of one or more molecules present
- provide quantitative information about composition of complex protein samples
- can also provide information about conformational properties

**Microscopy**

- used to examine to structure and formation of aggregates
- approaches include fluorescence (aggregates labelled with fluorescent probes) microscopy and electron microscopy (resolve oligomer structure at higher resolution)

**Seed amplification assays**

- aggregation assays that detect the presence of protein aggregates
- sample sonication and incubation with recombinant protein monomer
- aggregate seeds template and induce aggregation of the excess protein monomers
- reaction monitored by a thioflavin readout, aggregation curve characteristics recorded

**Extracellular vesicles protein measurement**

- released by cells, content represent central nervous system processes
- precipitation to increase concentration and neuronal enrichment with immune capture
- protein quantification with electrochemiluminescence (e.g. mesoscale discovery)

### Alpha-synuclein

Total, phosphorylated and oligomeric α-syn levels and their ratios in CSF, blood and other body fluids and tissues have all been explored for biomarker use (Table 1).

**Table 1:** Alpha-synuclein fluid and tissue biomarkers and their potential relevance to clinical trial design

Biomarker	Origin	Differentiating PD from healthy controls	Marker of disease severity	Differentiating PD from atypical parkinsonism	Predicting disease progression	Surrogate for disease progression
Total α-syn	CSF	–	+	–	–	+
	CSF (exosomes)	–	+	+	–	–
	Plasma/serum	++	−	+	–	–
	Plasma/serum (exosomes)	+++	++	++	+	+
	Saliva	+++	+	–	–	–
	Tears	++	−	−	–	–
	Skin	++	+	–	–	–
Ser-129p-α-syn	CSF	++	+++	++		+
	Serum/plasma	+	+	–	+	–
	Tissue/intestine	++	–	–	–	–
	Skin	+	–	++	–	–
Ratio of phosphorylated α-syn to total α-syn	CSF	+	–	–	–	–
	Saliva (exosomes)	+	–	–	−	–
Tyrosine phosphorylated α-syn	CSF	+	–	–	–	–
Tyrosine nitrated α-syn	Serum	+	–	–	–	–
Oligomeric α-syn	CSF	+++	++	+	–	–
	Plasma/serum/blood	+	–	–	–	–
	Serum/plasma (exosomes)	+	+	+	–	–
	Saliva	+++	−	+	–	–
	Saliva (exosomes)	+	−	–	–	–
Ratio of oligomeric to total α-syn	CSF	+++	+	+	+	+
	Plasma/serum (exosomes)	+	–	–	–	–
	Saliva	+	−	–	–	–
	Red blood cells	+	−	−	−	–
Oligomeric phosphorylated α-syn species	CSF	+	–	–	–	–
	Plasma	+	–	–	–	–
α-syn seed amplification	CSF	+++	+	+++	–	–
	Saliva	–	+	–	–	–
	GI biopsy	+	–	–	–	–
	Skin	++	–	–	–	–
	Olfactory mucosa	+	–	+	–	–

α-syn = α-synuclein; GI = gastrointestinal tract; PD = Parkinson’s disease. Grading approach adapted from Majbour *et al*.^20^

− = No effect (also scored if negative in a meta-analysis).

+ = Effect in 1 study/inconsistent results across studies.

++ = Effect in 2–3 studies using single site cohort.

+++ = Effect in ≥3 studies or multisite cohort (also scored if positive in meta-analysis).

#### Distinguishing Parkinson’s disease from other conditions

Total free α-syn levels have been explored in CSF, plasma/serum, saliva and submandibular gland tissue and are of no diagnostic value in PD.^21–30^ Measurement of total α-syn levels in extracellular vesicles (EVs) either in CSF,^31^ plasma/serum^32–39^ or saliva^40^ can distinguish PD from controls.^33–37,39,41–43^ Total α-syn levels in EVs derived from neurons can also distinguish PD from atypical disorders, though best distinction is achieved when α-syn levels are combined with levels of other proteins such as clusterin.^36,44^ Similarly, differences in α-syn levels in neuronal- compared with oligodendroglial-derived EVs shows promise for distinguishing PD from multiple system atrophy (MSA).^38^ Phosphorylated α-syn at serine-129 (Ser-129p-α-syn) levels are elevated in the CSF,^25,45–48^ serum and plasma^49–52^ of PD patients, although similar elevations are seen in atypical parkinsonian conditions, limiting specificity/diagnostic use.^53–56^ Elevated levels are similarly seen for Ser-129p-α-syn in skin.^30,57–61^ A predilection for Ser-129p-α-syn deposition in autonomic compared with somatosensory nerve fibres and proximal to distal gradients could be applied for improving the distinction between PD and MSA-parkinsonian type (MSA-P).^62,63^

Levels of α-syn oligomers are also increased in the CSF,^28,48,64–68^ plasma,^69,70^ red blood cells,^71,72^ saliva and tears^29,64,73–78^ of PD patients (although again with a few teams reporting contradictory findings^67,79,80^). Oligomeric CSF α-syn levels taken alone, however, have unsatisfactory diagnostic properties.^25^ Combining oligomeric α-syn and aggregated tau measurement in serum neuronal-derived exosomes seems to distinguish PD from tauopathies well.^81^ Reliable quantification and differentiation approaches between protein species (oligomers, fibrils and other aggregated forms) are currently lacking.^51,53^ Making these distinctions will be critical in improving the diagnostic performance of aggregated forms, considering unique patterns have been noted in different synucleinopathies.^82,83^ Ratios of Ser-129p-α-syn and/or oligomeric α-syn to total α-syn are elevated in PD and seem most promising in overcoming the limitations of individual markers for differentiating synucleinopathies.^45,46,54,55,66,68,84,85^

Seed amplification assays such as real-time quaking-induced conversion (RT-QuIC) and protein misfolding cyclic amplification (PMCA) are arguably the most important achievement in the field of biomarkers to date and will likely be the most useful diagnostic biomarker for trials. These techniques can amplify and detect minute amounts of aggregated α-syn in CSF.^10,86–88^ Studies comparing brain and CSF samples have demonstrated excellent performance for distinguishing PD from healthy controls (sensitivity and specificity: 90–100%),^4–11^ with comparable results for both seeding methods^7,10^ across laboratories.^10^ Assays can also distinguish PD from non-synuclein disorders such as progressive supranuclear palsy (PSP) and corticobasal syndromes (CBS),^11^ although the accuracy in distinguishing MSA from these conditions is poor (sensitivity: 4–82%), while studies exploring the use of α-syn SAA to distinguish MSA from PD have also reported variable findings.^87–91^ As differences in α-syn strains and therefore biochemical, morphological and structural properties of the final α-syn SAA reaction products underlie PD and MSA phenotypic heterogeneity, different outcomes may be explained by the fact that different chemical environments (SAA reaction mixes) can differentially influence the formation and growth of different strains. Protocols optimized for PD may not therefore work so well for MSA detection.^11,92^

In attempts to avoid lumbar puncture, the use of α-syn SAA has been explored using samples obtained through less invasive approaches. Increased α-syn skin seeding activity has been observed in PD (post-mortem and living) patients with excellent distinction from non-neurodegenerative cases,^93^ while aggregation rates using RT QuIC correlate with cognitive and motor status.^8^ Similarly, seeding activity in submandibular gland tissue of PD patients has been noted, although sensitivity (73.2% versus 100%) and specificity (78.6% versus 94%) for distinguishing PD from healthy controls varies between studies,^94,95^ while preliminary findings in saliva are promising.^96^ A recent report demonstrating the excellent ability of serum immunoprecipitation-based RT-QuIC for distinguishing PD from healthy controls may herald a new approach to diagnosing PD via a simple blood test, although lower detection rates in MSA, likely due to technical factors, will still need to be overcome.^97^ Similarly, the demonstration of seeding activity from pathological α-syn derived from plasma EVs is also promising.^98^ The use of less invasive samples will be ideal for trial recruitment (given feedback from patients regarding tolerability of submandibular gland biopsy) but will require demonstration of comparability with the high sensitivity and specificity achieved with CSF (although a recent meta-analysis suggested comparability between CSF and skin for diagnostic purposes^90,99^).

#### Predicting severity phenotypes and measuring progression

Total free α-syn levels do not correlate with disease severity and their ability to predict and track progression is also poor.^22,25,49^ EV total α-syn levels also predict and track progression in PD poorly.^31,32,35,36,100^

While Ser-129p-α-syn levels do seem to reflect disease severity^45,46,101^ and motor symptom progression,^102^ an inverse relationship in later disease (potentially as a result of extensive neuronal damage)^53,103^ makes its use as a progression biomarker challenging if applied to trials with long-term follow-up or involving patients with established disease. CSF and serum levels of a number of other phosphorylated α-syn species have also been explored, although preliminary findings are somewhat conflicting.^104–106^ A rostro-caudal pSer129-α-syn deposition gradient in the gastrointestinal tract of PD patients has also been noted, reflecting neurodegeneration in the myenteric plexus,^107,108^ although this may be a reactive physiological phenomenon.^109^ Disentangling reactive from pathological components will be important, as deposition may occur here earlier and therefore guide earlier treatment in early motor stages where diagnostic criteria have yet to be totally fulfilled.

Oligomeric CSF α-syn levels can also reflect PD severity and progression,^47,54,101,103^ despite some contradictory evidence,^110^ although previously highlighted limitations of differentiating aggregated forms need to be addressed. Longitudinal measurement of Ser-129p-α-syn and/or oligomeric to total α-syn ratios might detect effective treatment responses.^45,46,54,66,68,84,101^ Similar findings have also been observed when measuring these ratios in serum and salivary EVs, although this does not seem to offer additional value.^35,36,39,85,111,112^

Correlation of α-syn SAA with disease severity and progression is unclear, and specific kinetic cut-offs remain elusive, although quantification of α-syn SAA end products with oligomer-specific enzyme-linked immunosorbent assay (ELISA) may be helpful in this regard.^10,20,113^ Taken together, the best α-syn candidate biomarkers for diagnosing PD to consider for clinical trials are α-syn SAA. The ratios of Ser-129p-α-syn and or oligomeric α-syn to total α-syn can also helpfully differentiate between synucleinopathies^45,46,54,66,68,84^ and are credible markers for tracking progression.

### Alzheimer’s disease-like biomarkers

Amyloid-β (Aβ) peptides are cleaved from the amyloid precursor protein (APP) into the peptides Aβ_42_ and Aβ_40_, which can form extracellular amyloid plaques.^114,115^ Tau proteins comprise highly soluble isoforms, while their hyperphosphorylation contributes to the development of neurofibrillary tangles (NFTs).^116^ Amyloid plaques are abundant in the CNS alongside NFTs in Alzheimer’s disease (AD), while NFTs are characteristic of PSP and CBS.^117,118^

#### Distinguishing Parkinson’s disease from other conditions

Biomarkers reflecting tau and amyloid pathology can be measured in CSF and blood and include free and EV levels of total tau (t-tau), phosphorylated tau (p-tau) and amyloid peptide isoforms (Aβ_42_ and Aβ_40_). Higher CSF t-tau and decreased Aβ_42_ levels occur in tauopathies. This combination best distinguishes PD from CBS, although the relative rarity of CBS makes widespread testing in PD trials of modest value.^119,120^ Preliminary evidence suggests that ultrasensitive tau SAA may identify/exclude patients with tauopathies from PD at trial recruitment,^121^ although a combined assay with α-syn would be more ideal.

The combination of reduced Aβ_42_ and increased t-tau and p-tau levels is collectively termed ‘an AD-like profile’ considering its specificity for diagnosing the condition.^122^ This profile occurs in a larger proportion of synucleinopathy patients with prominent cognitive dysfunction, i.e. Parkinson’s disease dementia (PDD) and dementia with Lewy bodies (DLB).^123–125^ CSF AD-like biomarkers may, therefore, be useful for differentiating DLB from other parkinsonian disorders, although for some interventional trials this distinction may be somewhat arbitrary. Levels of t-tau and p-tau are increased in all parkinsonian disease groups and combining them with Aβ_42_ only usefully differentiates PD from frontotemporal dementia.^126^ Taken together, these findings suggest free blood levels of these markers are unlikely to be of diagnostic value in trials.

#### Predicting severity phenotypes and measuring progression

Tau and AD pathology commonly coexist in synucleinopathy patients^127^ and correlate with an acceleration in cognitive decline.^128,129^ PD patients with lower CSF Aβ_42_ levels at disease onset also have earlier appearance of cognitive impairment and more rapid conversion to PD-related dementia.^68,130,131^ The measurement of CSF Aβ_42_ could, therefore, be of prognostic value by reflecting brain amyloid content even prior to apparent clinical cognitive impairment.^132^

Although Aβ_42_ and tau can also be measured in blood, levels correlate poorly with cerebral pathology,^133^ potentially due to extra-cerebral sources such as platelets. Ultrasensitive immunoassay technologies such as immunomagnetic reduction (IMR) improve this,^134^ although correlation with cognitive function has been inconsistent.^126,135,136^ Similarly, total tau protein blood findings have been variable,^135,136^ potentially due to rapid changes in blood concentrations,^137^ although higher t-tau levels seem to correlate with lower cognitive performance.^138^

Aβ_42_ and tau can also be detected in EVs. While also not of diagnostic value, elevated levels in combination with elevated α-syn^139,140^ and lower serine phosphorylated insulin receptor substrate (IRS-p312), a marker of neuronal insulin resistance in blood EVs,^141^ predict worse motor and cognitive dysfunction progression phenotypes well. Larger replication studies of Aβ and tau in EVs are needed to better assess their validity for predicting cognitive dysfunction in PD before adoption for widespread use.

Measurement of other phosphorylated tau species (p-tau181, p-tau217 and p-tau231) in CSF and plasma can discriminate AD patients from cognitively unimpaired subjects and reflect cognitive measures and progression.^142^ P-tau181 levels have been studied in PD, and their ability to predict disease severity and cognitive decline has been mixed and, therefore, they cannot currently be recommended for trial use.^143–145^ Other tau species also show promise in AD and need further exploration in PD cohorts.

### Neuroinflammation

Immune cells in the CNS and in the periphery are involved in PD neurodegeneration.^146^ Measurement of cellular components and levels of inflammatory mediators have, therefore, been explored for biomarker purposes (Table 2 ). Glial fibrillary acidic protein (GFAP) is released from astrocytes into the bloodstream, and its level can be used to distinguish PD from healthy controls,^147,148^ while its ability to discriminate PD from other atypical parkinsonisms is unclear. The glial activation biomarkers YKL-40 (chitinase-3-like protein 1) and MCP-1 (monocyte chemoattractant protein-1) are increased even further in atypical parkinsonian patients compared with PD and can thus reliably discriminate tauopathies from synucleinopathies,^149,150^ although this is best achieved by combining them with a panel of non-inflammatory CSF biomarkers [area under the curve (AUC) = 0.95].^151^ Among PD patients, GFAP levels seem to predict the development of dementia.^152^

**Table 2:** Fluid and tissue biomarkers from aberrant pathways noted in Parkinson’s disease and their potential relevance to clinical trial design

Biomarker	Origin	Differentiating PD from healthy controls	Marker of disease severity	Differentiating PD from atypical parkinsonian disorders	Predicting disease progression	Surrogate for disease progression
**Neuroinflammation**						
Glial activation markers (YKL-40)	CSF	+++	–	+	–	–
Glial activation markers (MCP-1)	CSF	+++	++	+	–	–
GFAP	Serum/plasma	+	++	–	+	–
T-cell subtype level/ratios	Blood	+++	+++	−	+	–
Neutrophil lymphocyte ratio	Blood	+++	+	–	–	–
CRP	Blood	+++	+	–	+	–
Interleukin levels	Blood	+++	+++	−	++	–
TNF	Blood	+++	+++	–	+	–
Complement levels	Blood	−	+	–	+	–
Chemokine ligand 5/RANTES	Blood	++	++	–	–	–
**Lysosomal dysfunction**						
Glucocerebrosidase activity	CSF	++	+	–	+	–
	Blood	++	–	–	–	–
β-hexosaminidase	CSF	+	–	–	–	–
Cathepsin D	CSF	+	–	–	–	–
Glucosylceramide	CSF	−	–	–	–	–
	Plasma	++	–	–	–	–
	Serum	−	–	–	–	–
**Mitochondrial dysfunction**						
DJ-1	CSF	+	+	–	–	–
	Plasma/serum	−	+	+	–	–
Peroxisome proliferator-activated receptor *γ* coactivator 1α	Blood	++	+	–	–	–
Fibroblast growth factor 21	Serum	−	–	–	–	–
Growth differentiation factor 15	Serum	−	–	–	–	–
**Synaptic markers**						
SNARE complex	Plasma/serum (exosome)	+	−	–	–	–
SNAP25	CSF	+	–	–	–	–
Neurogranin	CSF	+++	++	−	−	–
β-Synuclein	CSF	−	+	−	–	–
GAP43	CSF	+	–	–	–	–
Contactin-1	CSF	+	–	+	–	–
Pentraxins	CSF	+	+	–	+	–
Neurotransmitter levels	CSF	+	–	+	–	–
Dopamine metabolites (HVA, DOPAC)	CSF	+++	+++	–	+	−
	Plasma	++	+	–	–	–
**Axonal damage (NfL)**	CSF	−	+	+++	++	–
	Plasma/serum	−	+++	+++	+++	–
	Plasma/serum (exosome)	−	+	–	–	–

DOPAC = dopamine, 3,4-dihydroxyphenylacetic acid; HVA = homovanillic acid; NfL = neurofilament light chain; PD = Parkinson’s disease. Grading approach adapted from Majbour *et al*.^20^

− = No effect (Also scored if negative in a meta-analysis).

+ = Effect 1 study/inconsistent results across studies.

++ = Effect in 2–3 studies using single site cohort.

+++ = Effect in ≥3 studies or multisite cohort (also scored if positive in meta-analysis).

**Table 3:** Outlines the range of imaging biomarkers and their potential relevance to clinical trial design

Imaging modality	Differentiating PD from healthy controls	Marker of disease severity	Differentiating PD from atypical parkinsonian disorders	Predicting disease progression	Surrogate for disease progression
Transcranial sonography	+	−	+	–	−
T_1_-weighted structural MRI	++	+++	+++	++	+++
Neuromelanin MRI	+	+	+	–	++
Iron sensitive MRI	+++	+	++	+	+++
Diffusion MRI	+++	++	++	++	++
MR spectroscopy	+++	++	++	–	–
Functional MRI	++	–	+	–	+
**PET/SPECT**					
Radionuclide	–	–	–	–	–
α-Syn	−	−	−	–	–
Dopaminergic	+++	+++	−	−	+++
Non-dopaminergic	++	++	–	++	–
Synaptic density	++	+	–	–	−
**Metabolic and network imaging**					
Glucose metabolism	+++	+	++	++	+
Neuroinflammation	+	+	−	–	–

PD = Parkinson’s disease; SPECT = single-photon emission computed tomography. Grading approach adapted from Majbour *et al*.^20^

− = No effect (also scored if negative in a meta-analysis).

+ = Effect 1 study/inconsistent results across studies.

++ = Effect in 2–3 studies using single site cohort.

+++ = Effect in ≥3 studies or multisite cohort (also scored if positive in meta-analysis).

Neutrophil-to-lymphocyte ratios (NLRs) are indicative of overall inflammatory status and are elevated in PD compared with healthy controls^153^ as is the proinflammatory lymphocyte profile (diminished T-regulatory and increased T-helper cell levels).^154–157^ The NLR has been negatively associated with presynaptic radionuclide striatal-binding ratios and positively associated with motor impairment,^153,158,159^ while a proinflammatory lymphocyte profile shift is associated with more severe motor and cognitive impairment,^160,161^ and an increase in Tregs expressing CD49d is linked to lesser motor impairment.^162^ Altered lymphocytes lead to and are in turn influenced by cytokines. Elevated C-reactive protein (CRP), interleukin (IL)-6 and IL-10 as well as tumour necrosis factor α and chemokine ligand 5 (CCL5, RANTES) levels have been noted in PD.^163–171^ Current evidence does not, however, suggest these markers would help in distinguishing PD from atypical conditions considering inconsistent findings between studies^156,172–174^ and small-to-intermediate effect sizes.^175^ Similarly, associations with non-motor symptoms noted particularly for IL-6 and IL-10^176^ are unlikely to be of value for trial design, although associations of pro-inflammatory cytokines, particularly CRP and CCL5, with reduced survival^177^ and the development of motor and cognitive impairment^178–180^ are of value for both prognosis and monitoring progression.

Taken together, the value of individual inflammatory markers is low, although combining several inflammatory markers to predict disease progression will likely contribute to future approaches.^180,181^ While better validated general biomarkers of progression exist, these panels could be particularly useful for enriching trials that test agents targeting inflammatory pathways.

### Genetics and gene regulation

The relationship between genetic risk factors for PD and the pathophysiological processes underlying PD are under renewed scrutiny based on the use of α-syn SAA in CSF. People with leucine-rich repeat kinase 2 (LRRK2) mutations may develop typical PD, positive α-syn SAA in CSF and typical PD pathology at post-mortem,^182^ while the phenotype, pathophysiology and α-syn SAA findings and post-mortem pathology can also be completely different, despite the same LRRK2 mutation.^183^ The far lower rates of positivity of the CSF α-syn SAA among *LRRK2* mutation carriers questions whether these patients should be included within trials specifically targeting α-syn and potentially other broad interventions being considered for PD neurodegeneration.^184^ Nevertheless, there is great interest in targeting LRRK2 as a means of influencing disease progression in PD, and genetic status may be of greater relevance for these interventions than other biomarkers. That said, the most advanced LRRK2 inhibitor trial has pragmatically chosen to focus recruitment of a combination of PD patients with and without *LRRK2* mutations (NCT05348785), while other LRRK2-specific interventions may specifically want to recruit the subgroup who are positive for the α-syn SAA.

Of relevance to this point, molecular dysfunction of pathways downstream from *LRRK2* also occur, and these are being explored as biomarkers in trials targeting this enzyme. pS1292-LRRK2 levels are higher in urinary EVs in idiopathic PD and correlate with motor severity.^185^ Furthermore, CSF EV pS1292-LRRK2 levels are 10-fold higher than urinary EV levels, suggesting relevance for CNS activity.^186^ Genetic variability may, therefore, be considered when selecting patients for precision medicine interventions as well as for helping to balance trial arms for progression or adjusting for baseline differences in longitudinal analysis. pS1292-LRRK2 levels or other downstream molecular abnormalities [whole-blood pS935 LRRK2 levels, peripheral blood mononuclear cell pT73 Rab10 levels, urine di-22:6-bis(monoacylglycerol) phosphate and CSF total LRRK2] may become useful tools for measuring target engagement and the therapeutic response to agents specifically targeting these pathways, as has been demonstrated in a recent early stage LRRK2 inhibitor trial^187^ (Supplementary Table 1).

Other genetic factors can also determine phenotypic severity and progression. PD patients with the A53T α-syn mutation experience worse autonomic and cognitive deterioration,^188^ while apolipoprotein E (APOE4) and glucosidase beta acid 1 (GBA1) PD patients have accelerated cognitive^189–193^ and motor deterioration,^194^ although this may be constrained to specific mutations/polymorphisms.^195–197^ Polygenic risk scores for predicting the rate of progression appear promising but need replication.^198,199^

Non-coding RNAs (ncRNA) contribute to gene expression regulation. Micro RNAs (miRNAs) are small ncRNAs (sncRNAs), which have been explored for biomarker potential. Unique serum miRNA patterns comprising upregulation (miR-6836-3p and miR-6777-3p) and downregulation (miR-493-5p, miR-487b-3p and miR-15b-5p) have been noted in PD^200,201^ and supported by known involvement of these miRNAs in PD pathogenic processes. Sampling, quantification and analysis approaches need to become standardized to facilitate between study comparisons. SncRNA analysis from CSF EVs may also be worth further exploration.^202^ While plasma EV miRNA measurement appears useful when distinguishing PD from healthy controls [AUC 0.85 (miR331-5p) and 0.90 (miR-505)^203^], the combination of miR153 and miR-409-3p using the CSF EV approach is even more impressive (AUC 0.99).^204^ miRNAs may likely play a diagnostic role in future trials depending on the mode of action of the drug being studied.

### Lysosomal dysfunction

The *GBA1* gene encodes the lysosomal enzyme β-glucocerebrosidase (GCase). GBA1 mutation carriers have almost uniformly positive α-syn SAA in CSF.^4^ Impaired GCase and other lysosomal enzyme activity [e.g. cathepsin D (CTSD)] in GBA1-carrier and non-carrier PD patients leads to lysosomal dysfunction, thus negatively impacting α-syn degradation.^205,206^ Although CSF GCase activity depends on the specific GBA1 mutation carried, levels are also lower in idiopathic PD patients compared with controls.^207^ GCase levels are, however, of low value for diagnosing PD, although combining GCase activity with oligomeric/total α-syn ratios (AUC = 0.87, 82% sensitivity, 71% specificity), as well as other lysosomal enzymes (CTSD and β-hexoxaminidase) and Aβ_42_, improves this (AUC = 0.83, 75% specificity, 84% sensitivity).^208^

CSF GCase levels correlate with cognitive impairment,^209^ while activity also seems to predict subsequent development of dementia regardless of genetic status.^210^ CSF GCase levels may, therefore, usefully allow enrichment of clinical trial arms testing agents targeting this enzyme (even in the absence of a GBA1 mutation) as well as a method for confirming target engagement. Blood GCase activity is also reduced compared with healthy controls, although prediction of progression has not been explored.^211,212^ GCase activity has been used as an exploratory outcome in recent disease modification trials in conjunction with its downstream hydrolytic product glucosylceramide (Supplementary Table 1). Glucosylceramide can distinguish GBA-PD from idiopathic PD and healthy controls and be measured in both plasma and peripheral blood mononuclear cells and therefore used as a biomarker for target engagement in clinical trials targeting GBA-PD.^213,214^

### Mitochondrial dysfunction

Mitochondrial dysfunction contributes to the pathogenesis of PD.^215^ The existence of inherited autosomal recessive parkinsonism due to mutations of parkin (PRKN), PTEN induced kinase 1 (PINK1) and the protein deglycase (DJ-1) gene, which encode proteins that mediate mitophagy, supports this link.^216,217^ Typical α-syn pathology is less consistently reported in people with these mutations, and the rate of positivity of the α-syn SAA in CSF is also low,^97,184^ thus reinforcing the potential importance of both genetic testing and selection of additional other biomarkers during trial recruitment and follow-up, depending on the mode of action of the agent being tested.

The best explored mitochondrial biomarker in this context is CSF DJ-1, levels of which are decreased in PD^218,219^ compared with controls and correlate with disease severity,^21^ although similarities with other parkinsonian syndromes make its diagnostic use unlikely.^220,221^ Similar poor diagnostic value has been noted for serum and plasma DJ-1 levels.^222–224^ Other less well studied biomarkers include phosphorylated ubiquitin at the serine 65 residue (pSer65Ub), which occurs by virtue of the loss of mitochondrial membrane potential triggering the stabilization of PINK1 at the outer mitochondrial membrane.^225^ While increased pSer65Ub levels have been observed in PD post-mortem brains, lower levels have been identified in familial PD with PINK1/parkin mutations.^226,227^ Investigations of this marker in biofluid samples will be of interest, possibly as confirmation of target engagement and longitudinally to assess progression rates of disease in these PD subtypes. Similarly, the peroxisome proliferator-activated receptor *γ* coactivator 1 alpha (PGC-1α) has been of interest due to its role as a regulator of mitochondrial function.^228^ The PGC-1α reference gene and PGC-1α levels are downregulated in human brain and blood leucocytes in PD compared with control patients, and this negatively correlates with disease severity.^229–231^ Interventions targeting mitochondrial processes might usefully measure peripheral levels of PGC-1α.

A concern, however, for the use of mitochondrial blood-based biomarkers is that they do not recapitulate ‘neuronal’ mitochondrial dysfunction. Genetic mutations leading to mitochondrial dysfunction in PD often show tissue-specific expression patterns, and therefore peripheral blood changes may lack interpretability.^232,233^ This is supported by a recent study showing negligible diagnostic value for well-established biomarkers of mitochondrial disease such as fibroblast growth factor 21 and growth differentiation factor 15 in reflecting mitochondrial dysfunction in PD patients.^226^

### Insulin resistance

The coexistence of type 2 diabetes mellitus (T2DM) with PD results in more rapid motor and cognitive progression.^234–237^ Faster progression appears to be independent from the existence of vascular disease in the brain^238^ and at least in part explained by disruptions in physiological brain insulin signalling (central insulin resistance)^239^ contributing to neurodegeneration.^240^

Central insulin resistance can be measured through abnormalities in insulin signalling mediated by insulin-receptor substrate-1 (IRS-1). Tyrosine IRS-1 phosphorylation (IRS-1 p-Tyr) evokes insulin responsiveness, while serine phosphorylation primarily deactivates IRS-1 and attenuates insulin signalling.^239,241^ Elevated IRS-1 phosphorylation at serine positions 616 (IRS-1 p-S616) and 312 (IRS-1 p-S312) represents attenuated insulin signalling^242,243^ and has been noted in plasma EVs of PD patients.^244,245^ Decreased IRS-1 p-Tyr distinguishes PD patients from healthy controls and predicts cognitive impairment and motor severity.^141^ Increases in EV IRS-1 p-Tyr were associated with motor benefits from exenatide in a clinical trial, while increases in downstream p-Akt S473 predicted treatment response (Supplementary Table 1).^244^

Peripheral insulin resistance as defined by a Homeostatic Model Assessment for Insulin Resistance (HOMA-IR) value ≥ 2.0 or glycated haemoglobin (HbA1c) concentration ≥5.7%, occurs in up to 60% of PD patients.^246^ The mechanistic importance of these finding in PD remains unclear, as the HOMA-IR is not associated with cognition or motor symptoms.^247,248^ Abnormal range HbA1c levels, however, predict motor and cognitive severity and progression in PD, while also being associated with the degree of axonal damage.^249–252^ Further exploration of insulin resistance and/or body mass index in the selection of patients for trials of agents that mechanistically target this pathway is clearly of potential importance, while measurement of central insulin resistance using exosome IRS-1 p-Tyr may turn out to be of utility in confirming target engagement for a growing number of agents currently being studied for disease modification.^253^

### Synaptic degeneration

Disruptions to vesicle-mediated trafficking and secretory pathways with downstream effects on neurotransmitter levels and signalling as well as synaptic plasticity are key features of synucleinopathies.^254^ Proteins at different levels of this process have been explored for biomarker use (Table 2). Evidence to date suggests limited usefulness in PD, in part due to the confounding effect of dopaminergic therapies. Despite some studies suggesting alterations in serum and CSF levels of synaptic dopamine potentiators [β-synuclein and growth associated protein 43 (GAP43)]^254–260^ and markers of synaptic plasticity [neurogranin (Ng), contactin-1 (CNTN-1) and the zinc transporter ZnT3] in PD, inconsistencies between studies and poor correlation with motor severity and cognitive progression make future utility unlikely.^259,261–268^

CSF concentrations of the secretory granule proteins (VGF and secretogranin-2) and the dense core vesicle protein prodynorphin are potentially useful in distinguishing PD from DLB or predicting cognitive decline.^269,270^ Similarly, preliminary studies suggest CSF levels of the excitatory-inhibitory regulatory protein, neuronal pentraxin-2 (NPTX2)^270^ and the glutamate receptor GluA3^262^ suggest value in reflecting cognitive status and distinguishing PD from DLB^271^ and thus warrant further exploration in the assessment of cognitive progression.

Measuring panels of CSF protein levels reflecting neurotransmitter secretion, synaptic plasticity and autophagy will likely shape any future use of these markers.^272^ An example of this approach includes combining CSF and serum EV levels of the principal components of the soluble *N*-ethylmaleimide sensitive factor attachment protein (SNARE) complex [synaptosomal-associated protein 25 (SNAP-25), the syntaxins 1A and 1B, syntaxin-binding protein-1 and the vesicle-associated membrane proteins (VAMP-1, VAMP-2)] with oligomeric α-syn to improve diagnostic accuracy.^111,263^ Similarly, combining CSF Ng, NPTX2, total α-syn and age^273^ or CNTN-1, total α-syn, total tau, phosphorylated tau and Aβ_1-42_^261^) can also improve diagnostic distinction.

A similar approach would also be worthwhile when considering the use of neurotransmitter metabolites. Despite decreased CSF levels of the dopamine metabolite homovanillic acid (HVA) being consistently noted in PD,^274–279^ repeated measurements in the Deprenyl and Tocopherol Antioxidative Therapy of Parkinsonism (DATATOP) study did not suggest usefulness for monitoring progression. Simultaneous metabolite panel measurement of dopaminergic [e.g. 3,4-dihydroxyphenylalanine (DOPA), dopamine, 3,4-dihydroxyphenylacetic acid (DOPAC)], noradrenergic (e.g. 3,4-dihydroxyphenylglycol, 4-hydroxy-3-methoxyphenylglycol) and serotonergic [e.g. 5-hydroxy-3-indoleacetic acid (5-HIAA)] metabolites in CSF,^278^ however, correlates better with motor severity and dopamine transporter single-photon emission computed tomography (DAT-SPECT) uptake,^280,281^ and utility of the panel as a progression marker needs to be further explored.

### Axonal damage

Neuro-axonal damage represents the end event in the pathophysiology of PD. Axon cytoskeletons are comprised of neurofilaments, structural proteins which allow for growth, with large, myelinated axons having the highest content.^282^ Neurofilament subunits are released upon axonal injury irrespective of the cause.^282^ The neurofilament light chain (NfL) subunit is of diagnostic value in degenerative parkinsonian syndromes,^283^ while also correlating with nigrostriatal degeneration and greater reductions in presynaptic putaminal DAT ratios over time.^284,285^ This said, CSF NfL concentration does not seem to be increased in early PD,^286^ and significant increases are more indicative of atypical diagnoses than PD.^283,286–288^

Blood NfL strongly correlates with CSF NfL^289–291^ and reflects neurodegeneration in PD.^291–294^ Although NfL levels were not elevated in a meta-analysis considering all patients with PD^290^ and in one study exploring EV NFL levels,^295^ levels seem to be higher in more advanced PD^289,291,293,296^ and the more severe postural instability and gait disorders (PIGD)-subtype.^297,298^ Consistent inverse associations with cognitive scores have been reported,^48,292–294,299–302^ while NfL levels also predict more severe motor progression,^285^ cognitive decline^298,303^ and progression to milestones [walking-aid, nursing-home living, reaching final Hoehn and Yahr (H&Y) Stage 5 or death]. Blood NfL may, therefore, be useful for trial stratification, although its potential use as a surrogate end point might depend on the disease stage of recruited participants and trial duration.^296,304^

The highest yield when using NfL seems to lie in combining it with clinical and disease-specific fluid biomarkers. Examples of this include the ratio of NfL to Aβ_42_ in CSF, discriminating PD from PSP with good accuracy (AUC 0.93, sensitivity 89%, specificity 93%)^305^ as well as the use of a stepwise approach of firstly distinguishing synucleinopathies from non-synucleinopathies with skin α-syn SAA and then further distinguishing MSA from PD with NfL^306^ or by combining CSF NfL, CSF α-syn SAA and brainstem imaging.^307^ Similarly, PD progression is better predicted when combining markers with serum NfL, genetic status (ApoE4 and GBA) and validated prognostic clinical variables (age, verbal fluency, Unified Parkinson’s Disease Rating Scale axial scores) predicting unfavourable progression better than individual markers.^296^

## Imaging biomarkers

A range of imaging modalities have been explored for their biomarker potential (Table 3). These include sonographic measurement of nigral signal, imaging approaches that measure brain structure, spectroscopy to explore brain biochemical changes, functional imaging to measure connectivity changes and radionuclide imaging to assess pre- and postsynaptic dopaminergic and non-dopaminergic integrity as well as metabolic functional changes (Box 2). Each approach has its strengths and weaknesses, and potential biomarker roles in trials will depend on the stage of disease being studied as well as practical considerations of availability and effect strengths alongside and in comparison with fluid biomarkers.

Box 2 **Biomarker imaging techniques**

**Transcranial sonography**

- ultrasound echogenicity measurement of brain tissues or structures through intact cranium—limited by lack of bone window in some subjects and inter-technician variability

**Structural MRI**

- quantification of brain structural change using regions-of-interest or whole-brain approaches
- commonly used sequences include T_1_, T_2_, T_2_*, R_2_* (R_2_* = 1/T_2_*)-weighted, susceptibility-weighted, proton-density-weighted, fluid-attenuated inversion recovery and neuromelanin-sensitive approaches

**Proton magnetic resonance spectroscopy**

- estimates relative concentrations of proton-containing metabolites in brain
- metabolites commonly assessed include *N*-acetylaspartate, choline-containing compounds, myo-inositol and creatine

**Functional MRI**

- evaluates neuronal activity by measuring transient variations in blood flow and variation correlation in functionally connected regions
- utilized under task-based or under resting-state conditions

**Radiotracer imaging**

- measures pre- and postsynaptic receptor and transporter density as well as glucose metabolism and microglial activation using different radiotracers
- provides information on nigrostriatal dopaminergic, serotonergic and cholinergic system integrity, regional tissue glucose metabolism and activity and status of microglial-mediated inflammation

In the proposed staging system for PD, the development of dopaminergic dysfunction has been incorporated as an important staging threshold.^12^ The range of imaging approaches that could be used for this are variable in their ability to discriminate PD from other pathophysiological processes as well as their potential for measuring the rate of progression of PD.

### Transcranial sonographic imaging

Increased substantia nigra (SN) echogenicity, likely due to accumulation of nigral iron, is observed in PD,^308–310^ although a proportion of healthy controls and essential tremor patients also exhibit this.^311^ This sign can, however, differentiate PD from PSP and MSA with good sensitivity (91%) and specificity (82–96%).^308^ Hyper-echogenicity remains unchanged over follow-up^312^ and does not correlate with disease severity^310,313^ or presynaptic DAT loss,^314^ thus limiting use as a progression marker.

### Structural MRI techniques

Structural MRI approaches comprise; T_1_-weighted structural imaging methods, which measure cortical and subcortical volumetric changes and brain atrophy; neuromelanin-sensitive T_1_-weighted imaging, which is sensitive to measuring neuromelanin-iron complexes; iron-sensitive MRI which captures iron deposition and dopaminergic cell loss; and diffusion imaging using either single-tensor or two-compartment diffusion modelling (free-water), which reflects neurodegeneration and/or neuroinflammation.

#### T_1_-weighted structural MRI

T_1_-based structural MRI methods comprise cortical thickness measurement, voxel-based morphometry (VBM) and deformation-based morphometry (DBM). Differences in these approaches are summarized in Box 2.

Structural differences in the midbrain, putamen, brainstem and cerebellum can distinguish PD from atypical parkinsonian disorders.^315^ This distinction is, however, best made in later disease stages at a time when disease modification approaches may be hardest to achieve. Novel automated indexes may improve this though will need to be tested in independent cohorts.^316^

In the PPMI cohort, deformation-based morphometry detected a unique atrophy pattern, which predicted motor progression in early PD without dementia.^317^ A faster decline in prefrontal and cingulate cortices and the caudate and thalamus has also been seen in *de novo* PD compared with controls,^318^ while greater frontal atrophy after 18 months has also been noted in PD patients without cognitive impairment with a disease duration of only 2 years^319^ (though these findings were separately contradicted^320^).

Studies in individuals with moderate to late-stage PD without dementia have also varied. No VBM differences were noted in one study,^321^ while another found reduced grey matter in the frontal lobe.^322^ Longitudinal atrophy of occipital and fusiform regions has been noted in patients with a disease duration of over 5 years without cognitive impairment, while patients with cognitive impairment develop greater and more widespread atrophy in supplementary motor area, temporal, parietal and occipital cortices.^323^ Accelerated loss of gyrification in bilateral frontal and parietal regions in patients with a disease duration greater than 5 years compared to less than 5 years has also been noted.^324^

In summary, T_1_-weighted structural MRI methods are sensitive to neurodegenerative progression even in the absence of cognitive impairment, although this also seems to be better in more advanced disease stages. Replication studies demonstrating patterns of atrophy progression depending on disease stages are however currently lacking and will be important before recommendation for trial use. Furthermore, ascertaining the precise role of ultra-high-field scanners (7 T and above), which can provide sub millimetric anatomical information and higher degrees of diagnostic detail compared with 3 T MRI,^325^ will be important. Planned future longitudinal studies will be critical for informing this.^326^

#### Neuromelanin and iron sensitive imaging

Neuromelanin imaging (NMI) demonstrates only moderate sensitivity and specificity for distinguishing PD from healthy controls,^327–331^ while signal differences are also suboptimal for distinguishing atypical parkinsonian conditions from PD.^332,333^ In contrast, however, NMI shows reduced signal across disease stages (disease duration of 1.5–10 years) with a ventrolateral to anteromedial SN progression pattern consistent with the neuropathological patterns of cell loss.

Iron-sensitive techniques including R2* relaxation imaging, susceptibility-weighted imaging (SWI) and quantitative susceptibility mapping (QSM) have similar ability to quantify nigral iron deposition as NMI.^334–336^ The absence of dorsal nigral hyperintensity corresponding to the region of nigrosome-1 (DNH) on iron-sensitive sequences distinguishes PD from controls well^325,337,338^ regardless of disease duration.^339^ Use for distinguishing atypical disorders from PD is however lacking, while progression marker use seems to be disease duration-dependent.

Although striatal, nigral, globus pallidus and caudate R2* relaxation rate increased in two separate studies after 2 years in early-stage PD,^335,340^ separate studies exploring R2* or QSM in *de novo* patients^336^ and patients with a disease duration <1 year showed no longitudinal changes.^339^ The use of R2* as a progression marker becomes clearer, however, in later disease stages,^339^ with increased relaxation time in SN R2* mapping over 3 years correlating with motor severity in cases with an initial disease duration of 5 years,^341^ while faster progression in the SN pars compacta seems to occur after a disease duration >5 years.^339^

Taken together, NMI and iron-sensitive imaging could potentially be developed usefully as progression biomarkers, although values will need to be considered in the context of disease duration. Obviously, the use of iron-sensitive modalities will be particularly advantageous in trials targeting iron.

#### Diffusion imaging

Although some studies have demonstrated reduced SN fractional anisotropy with single tensor diffusion imaging in early PD,^342–344^ this was not confirmed by a meta-analysis of 10 studies.^345^ Evidence in later disease (disease duration 10 years) is limited to one study demonstrating more anterior and rostral SN involvement.^344^ On balance, this approach cannot currently be recommended for progression marker use. The finding of diffusion abnormality of the nucleus basalis of Meynert predicting development of cognitive impairment could be explored for balancing arms in small trials or selecting phenotypes that are likely to respond to specific treatments though replication of this finding is important.^346^

Free water imaging studies have been more consistent with increased signal in the posterior SN being noted in early PD.^347,348^ Free water in the posterior SN also increases over 4 years and changes over 1 year can predict the H&Y 4-year change.^348^ This increase continues in later disease stages (duration over 7 years), where longitudinal increases in free water occur in the anterior but not posterior SN.^349^ This modality is promising as a progression biomarker but may require selection of the region of interest depending on the disease stage. Free water imaging of the basal ganglia, midbrain and cerebellum and the application of automated imaging differentiation is promising for differentiating PD from atypical conditions.^350^ This approach was found to be superior to a conventional magnetic resonance Parkinsonism index as well as plasma NfL levels for distinguishing PD from atypical conditions.^351^

### Proton magnetic resonance spectroscopy

Proton magnetic resonance spectroscopy (MRS) reveals the metabolic status of the region sampled for a specific disease process. In PD, *N*-acetyl aspartate/creatine (NAA/Cr) ratios in the SN are reduced compared to controls and correlate with disease severity.^352,353^ Lower ratios have also been noted in the lentiform nucleus (LN), temporoparietal and posterior cingulate cortices, as well as the presupplementary motor area,^354–357^ although correlation with disease severity is less clear.^355,356^ NAA/Cr ratios are lower in the rostral SN in PD with an inverted pattern in atypical parkinsonian patients and healthy controls.^358^ Taken together, there is some preliminary level of evidence that MRS could serve to improve PD diagnostics, but it may be best used in combination with conventional MRI to increase specificity.

Phosphorus based MRS (^31^P-MRS) has been of specific interest for a subset of potential interventions as it can assess mitochondrial function. *In vivo* Pi/ATP and PCr/ATP ratios reflect oxidative phosphorylation pathways.^359^ Reductions in ATP and PCr^360^ and increased Pi/ATP ratios^361^ in the putamen and midbrain of PD patients compared with controls have been reported while differences can also distinguish PD from PSP (AUC 0.93).^362^ Longitudinal ratio improvement suggestive of target engagement was also noted in a recently completed disease modifying trial of ursodeoxycholic acid.^363^

### Functional MRI

Resting-state and task-based functional MRI reveal networks involved in motor, cognitive and affective processes. Network impairments have been associated with motor and non-motor symptoms. Reduced resting-state connectivity between the striatum and thalamus, midbrain, pons and cerebellum has been noted in PD as have connectivity changes between cortical and subcortical areas.^364^ Reduced resting-state functional connectivity within the basal ganglia network can differentiate PD from healthy controls (sensitivity 100%, specificity 89.5%),^365^ while cerebellar connectivity with multiple brain networks differs between PD and MSA.^366^ Longitudinal task-based functional MRI can track progression with declining activity in the putamen and primary motor cortex over 1 year,^367^ although the impact of levodopa administration on network connectivity is an important consideration.^368^ While the available evidence for this modality is promising overall, more widespread replication of diagnostic and progression findings are necessary.

### PET/SPECT imaging

#### Radionuclide imaging

Several radiolabelled probes for imaging α-syn have been explored though no tracer is currently of diagnostic value for PD. Issues to overcome include developing tracers for intracellular targeting with ideal lipophilicity and tracer selectivity for α-syn over amyloid and tau aggregates.^369,370^ More recently, however, a newly developed α-syn PET tracer, ^18^F-ACI-12589, was shown to bind to basal ganglia and cerebellar white matter in a small cohort, although this was confined to MSA patients.^371^ Larger studies examining diagnostic accuracy for distinguishing PD from MSA will be critical.

#### Dopaminergic tracers

A variety of radionuclide tracers are available to examine pre- and post-synaptic striatal dopaminergic function using PET or SPECT imaging. At the presynaptic level, molecular targets and their respective tracers include L-aromatic amino acid decarboxylase [AADC/tracer fluorodopa (F-DOPA)], vesicular monoamine transporter 2 (VMAT2/tracer ^11^C-dihydrotetrabenazine) and the DAT (DAT/tracers CFT PET and ^123^I-CIT SPECT) density.

These markers are sensitive for the detection of dysfunction or loss of striatal dopaminergic terminals and enable the identification of parkinsonian syndromes with nigral neurodegeneration but do not reliably distinguish PD from atypical disorders. Visual assessment for the presence of nigrostriatal degeneration with this modality is increasingly used in trial recruitment^372^ to exclude patients with clinical presentations in keeping with PD but with scans without evidence of dopaminergic deficit (SWEDDS) due to e.g. drug induced parkinsonism.^373–375^ Objective measurement of striatal uptake in comparison to other regions may, however, be more useful in trials recruiting patients with more established PD as these ratios can reflect motor and non-motor disease severity as well as progression through disease stages, although hemispheric dominance and type of tracer used are important considerations.^376^ Striatal dopaminergic markers decline most prominently in the first years of motor disease before largely plateauing within 5 years of diagnosis.^377–380^ Quantification of dopaminergic markers in the midbrain/SN may be better markers beyond this point.^381^

The type of dopaminergic tracer used can potentially be critical for tracking progression in trials and measuring treatment response with VMAT2 imaging is less subject to compensatory changes in expression than DAT and F-DOPA.^382^ Quantitative dopaminergic assessments have been used in a number of recent disease modification trials though with overall negative findings to date (Supplementary Table 1).

Dopamine receptor expression can also be estimated at the post-synaptic level with PET ligands such as ^11^C-raclopride, ^18^F-fallypride or ^123^I-IBZM SPECT (all of which bind to D2 receptors) or agents such as ^11^C-NNC 112, which binds to D(1) receptors.^383^ Preservation of post-synaptic dopamine receptors is typical of PD whereas post-synaptic receptor loss early in the disease is more likely indicative of an atypical form of parkinsonism. Imaging results depend on the dose and timing of oral dopaminergic replacement and the usefulness of this type of imaging approach may perhaps be restricted to restorative approaches such as cell or gene therapy interventions.^384^

#### Non-dopaminergic tracers

Radionuclide imaging studies of the serotonergic and cholinergic systems demonstrate associations with non-motor PD pathophysiology. Reduced binding on serotonergic imaging has been noted in individuals with early PD (disease duration less than 5 years).^385^ Serotonergic denervation also correlates with increased dopamine turnover and reduced levodopa responses.^386^ In later disease stages (disease duration 5–10 or more years), serotonergic transporter binding remains reduced compared to controls,^385^ and the degree of serotonergic pathology is associated with cognitive decline.^387^ Cholinergic denervation also occurs in early PD (disease duration less than 3 years) but is more pronounced in PD with dementia.^388^ Noradrenergic activity, quantifiable by PET imaging is reduced in PD and is associated with the presence of RBD and cognitive impairment.^389^ The utility of these markers in tracking progression is of interest but not yet sufficiently clear.

#### Synaptic density

Synaptic density quantification irrespective of neurotransmitter type has also been of interest in PD. Tracers quantifying the concentration of the synaptic vesicle 2A protein (^18^F-UCB-H or ^11^C-UCB-J) reflect this and have been studied in several cohorts. Lower binding potential in both cortical and subcortical regions have been noted in PD though this is most prominent in the SN.^390^ Correlation with clinical status has, however, been inconsistent though one study suggested more prominent and extensive reductions in PD dementia and DLB cases.^391–393^ Similarly, small cohort studies using ^11^C-UCB-J PET did not note binding changes over 2 years.^391,394^ Current evidence therefore does not support the use of this marker in clinical trials.

### Metabolic and network imaging

#### Glucose metabolism

^18^F-FDG-PET parieto-occipital hypometabolism is noted in PD,^395,396^ while preserved glucose metabolism in the basal ganglia distinguishes PD from MSA and PSP.^395^ Inferior parietal and left caudate glucose hypometabolism in PD also correlates with motor and cognitive deficits.^397^ A unique PD-related pattern (PDRP) characterized by elevated pallidothalamic and pontine metabolic activity with reduction in the supplementary motor area, premotor cortex and parietal association areas has also been noted in cases prior to dopaminergic treatment^398^ and can differentiate PD from atypical parkinsonism.^399^

PDRP progresses in early PD (disease duration less than 2 years) over 24 months, suggesting potential progression marker use in the early stages,^400^ although a critical limitation is that acute dopaminergic treatment diminishes the pattern.^401^ A PD-related cognitive pattern (PDCP) characterized by a reduction in the medial frontal and parietal association regions and metabolic increase in cerebellar cortex and dentate nuclei^402^ has also been described. This pattern seems to occur years after the PDRP,^400,403^ increases over time^400^ and is higher in those with dementia.^404^ The PDCP also correlates with memory and executive performance,^402^ while its lack of change with dopaminergic treatment potentially supports its use as a marker of cognitive dysfunction.^405^ These separate metabolic networks could potentially be used to track progression and treatment response in the appropriate setting.

#### Neuroinflammation imaging

The PET ligands ^11^C-PK11195, ^11^C-PBR28 and ^18^F-FEPPA, which bind to the 18 kDa translocator protein (TSPO) on mitochondria in microglia, have been used for imaging neuroinflammation with TSPO upregulation suggesting microglial activation.^406^

PD clinical severity and putaminal presynaptic dopaminergic integrity correlates with ^11^C-PK11195 binding.^407^ Binding affinity can vary with TPSO genetic polymorphisms which needs appropriate adjustment in analyses.^406,408^ Taken alone, TPSO patterns lack the ability to distinguish parkinsonian conditions though their future use may be as biomarkers of therapeutic response for interventions targeting neuroinflammation.^409^

## Limitations of biomarkers

A framework for considering the definition of PD according to the presence/absence of α-syn SAA-CSF is potentially a major step forward in planning PD trials. Several practical obstacles need to be considered however prior to the routine use/reliance on biomarkers in the clinical trial context. Firstly, acquiring some biomarkers, e.g. CSF, requires an invasive procedure, which may be unacceptable for some participants. Growing evidence of the equivalence of α-syn SAA-in skin to that seen with CSF could, however, overcome this limitation. The demonstration of equivalence of testing on even less invasive samples such as serum/plasma or within peripherally obtained EVs is therefore a priority. With greater demonstration of validity, routine testing of peripherally acquired biomarkers can become normal practice, for example the widespread availability of plasma NfL testing in healthcare laboratories.

Interpretation of discrepant results between studies attributable to preanalytical and analytical confounders, different techniques employed and a lack of factoring of different protein species measured (total α-syn versus oligomeric) needs careful critique. Similarly, imaging studies are affected by methodological discrepancies, including different assumptions for correction of serial data as well as sample size, power and study design caveats and the use of different outcome measures. Collaborative studies allowing analysis of larger sample sizes with adequate follow-up that employ standardized sampling and analysis methodology will improve these limitations, as demonstrated by the harmonization of large numbers of samples processed in the PPMI.

The major limitation in biomarker discovery is undoubtedly difficulty with validation. Association between a change in a biological assay alongside a clinical state need not equal causation. For example, biological changes may represent healthy compensatory responses to a pathological process. Furthermore, even biomarkers that do reflect active processes of neurodegeneration may not have linear relationships over the course of disease particularly if production ultimately declines because of widespread tissue death. While it is possible to use clinico-pathological data for validation, confirmation that a biomarker predicts slowing of disease progression necessarily requires the identification of an agent which achieves this according to our threshold, whether that be clinical, patient reported, functional impairment or quality of life milestones, which have inherent limitations.

To date, no single biomarker can yet be recommended to act as a surrogate for clinical disease progression in PD. Combinations of fluid biomarkers invariably increase the strength of their individual predictive properties. While fluid and imaging biomarkers are often collected from the same trial participants, explorations of the utility of multiple fluid biomarkers as a panel alongside imaging in combination are rare. This approach was partly adopted in the recent deferiprone trial (Supplementary Table 1) where brain iron content using T_2_* sequences and plasma ferritin and prolactin levels were used as combined markers of target engagement and specific measures of treatment effect while structural imaging for measurement of brain atrophy and DAT-SPECT imaging was used to explore the impact of the agent on overall disease progression (atrophy and nigrostriatal degeneration). Although clinical worsening in the deferiprone treated group complicates interpretation of how well the panel of biomarkers performed, one could argue that they did reflect the effect of the drug with decreased nigrostriatal iron content and plasma ferritin and increased plasma prolactin in the deferiprone group, while no inverse correlation between brain-structure volumes and iron content was noted in keeping with the negative clinical findings over a relatively short duration of follow-up.

Challenges for future trials will be in the choice of selection of suitable combinations of fluid and imaging biomarkers that complement each other. This will certainly need to be strongly guided by the biological action of the agent being tested and the stage of the disease of their participants being treated, although those biomarkers that appear to align most closely with disease progression should be prioritized. How much weight each biomarker in the panel will ultimately carry will become more easily evident following a positive clinical trial.

## Conclusions and recommendations

The identification of a better framework for the certainty of a PD diagnosis based on positivity of α-syn SAA-CSF is a major step forward, and less invasive equivalent alternatives will help even more. The further development of reliable biomarkers of PD neurodegeneration could further facilitate prognostication, identification of disease subtypes, conduct of clinical trials and identification of agents that may slow down or stop these processes. The precise role for biomarkers will depend on the mechanism of action of the agent in question, and the decision made regarding the stage of the illness at which the intervention is being applied. There is interest in recruiting people earlier in the neurodegenerative process, even prior to symptom onset, given that, intuitively, earlier intervention may provide a better chance of preventing irreversible cell death.^410^ Alongside trials in prodromal cohorts, there will remain a need to identify whether any disease modifying intervention has an impact on the 6–10 million people already struggling with symptoms and in need of prevention of further decline.

In this group, PD diagnosis is less difficult though a sizeable proportion of cases at this stage with atypical parkinsonian disorders can be mistaken as suffering from PD and therefore inadvertently recruited into disease modifying trials. While there will remain healthy debate whether α-syn oligomeric seeding and propagation is the primary cause of PD neurodegeneration, it appears that the α-syn SAA-CSF assay reflects an α-syn-related neurodegenerative process and can reliably distinguish synucleinopathies from other causes of parkinsonism/tremor with high specificity.

PD subtyping is also a high priority for better selection of responders. For example, interventions that specifically target an aspect of disease pathophysiology associated with genetic abnormalities could be specifically tailored to these patients.^411^ Mutations in GBA1 confer a worse prognosis and therefore a trial enriched with these patients may potentially allow an earlier signal of efficacy. In parallel, enhancement of GCase activity may also have therapeutic benefits in PD patients without GBA1 mutations.^412^

Features that strongly predict subsequent disease progression need to be carefully considered during treatment allocation. The randomization process itself should lead to balancing of features between placebo and active treatment arms, however this can fail to achieve this in smaller sized trials. The application of a panel of biomarkers for example pro-inflammatory immune markers which predict faster progression^180^ and reflect different aspects of disease-related pathways would be a useful approach to stratify patients into prognostic groups and potential responders to the treatment being tested, which will in turn enable more efficient and cost-effective collection of data and increase the likelihood of detecting an effect.

The most useful function of biomarkers is in the prediction that a change in any such biomarker reliably predicts slowing down of the neurodegenerative process that translates to reduction in disability accrual, and maintenance of function and quality of life. Towards this, the ratio of phosphorylated or oligomeric α-syn to total α-syn in CSF appears to be an encouraging fluid biomarker for disease progression. Technical challenges notwithstanding, measurement of one or both of these ratios may become routine practice in clinical trials of disease modifying agents to further improve diagnostic precision at baseline, minimize difference between trial arms and monitor changes in response to the intervention. The selection of a single fluid biomarker is likely to be a lower sensitivity surrogate for disease progression than the use of a panel of biomarkers. The development of a poly-biomarker, analogous to a polygenic risk score, will require careful modelling in large cohorts that have collected identical panels using agreed standardized operating procedures for their collection.

There are several structural imaging techniques that seem to track disease progression in PD reliably, perhaps the most useful of which are neuromelanin or free water MRI. Whether these allow sufficient resolution to quantify changes over shorter time periods than needed for conventional clinical methods, requires further data. Functional or PET imaging may allow more rapid confirmation of target engagement in trials, and their routine use may depend on the putative mechanism of action of the intervention, e.g. TSPO PET, in a trial of a neuroinflammatory intervention. While stabilization of fluid, imaging or tissue biomarkers should mirror attenuation of α-syn aggregation within the brain, it remains to be seen whether change in biomarker activity can reliably predict subsequent clinical disease progression.

In terms of recommendations, during the design and conduct of a clinical trial of a disease modifying intervention in PD, we suggest:

- For broad interventions, investigators should routinely collect a biomarker (CSF, skin, blood) that can be used for an α-syn SAA as part of the trial inclusion criteria. Currently, SAA offers the highest specificity in distinguishing PD from controls or PD-like conditions, but it’s utility in differentiating PD from MSA requires further assay refinement.
- For precision interventions, investigators should consider whether the planned intervention targets an alternative process that can be defined by an alternative genetic marker (LRRK2, GBA1, mitochondrial mutation) or measurable pathophysiological process (neuroinflammation, bioenergetics), irrespective of α-syn SAA.
- Investigators should consider incorporating such a biomarker within the trial inclusion criteria, while also ensuring the biomarker is appropriate for the stage of disease being studied.
- Where appropriate, the same biomarker might also be used to confirm target engagement of the intervention.
- Clinical outcome analyses may need to incorporate baseline differences in panels of wet biomarkers as well as imaging differences between treatment groups predictive of more rapid progression.
- Investigators should formally evaluate the relationship between biomarker changes and predicting the clinical effect of the intervention.
- Consideration should be given at an early stage how biomarker data can be usefully shared/integrated to maximize learning across interventions.

Until we have identified an agent that slows down clinical progression, it will be difficult to conclude the validity of any biomarker at predicting such disease modification. It appears as a somewhat circular argument therefore, that we need success, before we can be confident in our tools designed to help achieve success. Faced with this challenge, the most practical path forward is to systematically collect specimens from participants in clinical trials for future research, while also incorporating longitudinal measurement of encouraging biomarkers for continued comparison with clinical progression measures. This requires a degree of consensus in the PD trials community regarding standardized protocols for specimen collection and analysis. The Critical Path for Parkinson’s (CPP) consortium is helping to achieve this.^413^ Differences in the longitudinal change in biomarkers according to candidate interventions will undoubtedly help in the understanding of target engagement and help in the eventual prediction of long-term outcomes and ultimately are likely to become reliable surrogate outcome measures.

In conclusion, we should remain optimistic that the use of a combination of fluid, tissue and imaging biomarkers may become sufficient to reliably demonstrate disease modification. There is already a precedent that change in an imaging biomarker has been considered sufficient evidence, by some, to conclude disease modifying properties of aducanumab in AD.^414^ This decision has been controversial, and it is likely that a more robust conclusion in PD would only be reached once any combination of biomarkers has been comprehensively validated in relation to patient reports of clinical symptoms of relevance to their health and wellbeing. In the meantime, the best biomarker candidates can already likely improve the selection of participants and may contribute to early assessments of target engagement and of efficacy in counteracting pathophysiological mechanisms. An ongoing systematic process of confirming clinico-biomarker validity and utility is required.

## Supplementary Material

awad265_Supplementary_Data
