# Supplementary material for: How should we be using biomarkers in trials of disease modification in Parkinson’s disease?
Source: Brain. 2023 Aug 3;146(12):4845–69. doi: 10.1093/brain/awad265 (PMC10690028; doi:10.1093/brain/awad265)
Supplement: awad265_Supplementary_Data [file awad265_supplementary_data.pdf]

Supplementary Table 1 Summary of completed drug trials for disease-modification in Parkinson's disease that have explored the use of biomarkers.

**Molecular target**	**Mechanism of action**	**Drug(s)**	**Biomarker supporting target engagement**	**Biomarker relationship with primary outcome**	**Biomarker result**
Alpha-Synuclein	Inhibition of α-synuclein aggregation (autophagy-cAbl inhibitors)	Nilotinib[1] NCT02954978	CSF total & oligomeric alpha-synuclein & ratio, Dopamine metabolites (DOPAC, HVA) in CSF & Plasma, CSF total tau and P-tau 181, CSF-triggered receptors on myeloid cells levels, plasma	No correlation specified	Small amounts of nilotinib detected in the CSF, plasma DOPAC, but not CSF DOPAC or HVA level increased, reduction of α-synuclein oligomers, reduction of p-tau 181, decreased P-tau 181/total tau

		Nilotinib[2] NCT03205488	and CSF pan-tyrosine Abl, and plasma and CSF tyrosine 412 Abl (activity)		
			Nilotinib levels (CSF & serum) and dopamine metabolites (catecholamine catabolites and pre-cursors, serotonin & metabolite) in CSF	No correlation specified	Poor CSF penetration (half-maximal inhibitory concentration), No change in dopamine metabolites
	Anti-α-synuclein antibody	Prasinezumab[3] NCT03100149	DaT-SPECT Striatal Binding Ratio (SBR) in Putamen Ipsilateral to	No correlation specified	No DaT-SPECT change, 4 had antidrug antibodies

			Clinically Most Affected Side, Blood Anti-Drug Antibodies		
		Cinpanemab[4] NCT03318523	CSF α-synuclein seeding amplification assay to confirm presence, DaT-SPECT imaging	No correlation specified	Plasma levels demonstrated dose proportionality, No DaT-SPECT change
		Affitope (PD01A) Active alpha synuclein immunization (NCT01568099, NCT01885494,	Blood & CSF PD01A specific antibody and total α-synuclein levels.	Nil (Safety & tolerability)	Detected vaccine-triggered antibodies in blood and CSF, trend toward lower levels of alpha-synuclein in blood and CSF

		NCT02216188, NCT02618941)			
**LRRK2**	LRRK2 inhibitors (promoting autophagy)	DNL201[5] NCT03710707	CSF & plasma drug levels of drug, LRRK2 pS935& pRab10 in peripheral blood	Nil (Safety & tolerability)	LRRK2 pathway engagement and alteration of downstream lysosomal biomarkers, robust CSF penetration
	Antisense oligonucleotide for LRRK2 inhibition	BIIB094 [6] NCT03976349	Serum drug concentration, whole-blood phosphorylated serine 935 LRRK2, peripheral blood	Nil (Safety & tolerability)	CSF/unbound plasma concentration ratio~1; Whole-blood phosphorylated serine 935 LRRK2 (≤98%), peripheral blood mononuclear cell phosphorylated threonine 73 pRab10 (≤93%), cerebrospinal fluid

			mononuclear cell phosphorylated threonine 73 pRab10, cerebrospinal fluid total LRRK2, urine bis (mono-acylglycerol) phosphate		total LRRK2 (≤50%), and urine bis (monoacylglycerol) phosphate (≤74%)
**Lysosomal function**	Modulator of lysosomal enzyme β-glucocerebrosidase (GCase) activity	Ambroxol[7] NCT02941822<br><br>*GBA status explored as baseline predictor	CSF drug levels, GCase activity and protein levels and α-synuclein levels	No correlation specified	Drug detected in CSF, GCase activity decreased, GCase protein levels increased, α-synuclein concentration increased<br>*GBA status no impact
		LTI-291[8] NTR6598 (SAD) and NTR6705 (MAD)	CSF levels of drug and glyco-sphingolipids	Nil (Safety & tolerability)	Favorable pharmacokinetics, No glycosphingolipid changes<br><br>*GBA status no impact

		*GBA status explored as baseline predictor			
	Substrate reduction	Venglustat[9] NCT02906020<br><br>*only GBA mutation carriers recruited	CSF & plasma drug, glucosyl-ceramide & glucosyl-sphingosine levels	No correlation specified	Dose proportional drug increase, Glucosylceramide decreased in treated group in CSF & plasma
**Inflammation**	Altered T-cell lineage	Sargramostim[10] NCT01882010 NCT03790670	Immune phenotype and function, DNA methylation status, and gene and proteome analyses	No correlation specified	Blood Treg numbers, function, and hypomethylation of upstream FOXP3 DNA elements increased
	Enzyme myeloperoxidase (MPO) inhibition	AZD3241[11] NCT01527695	Plasma drug concentration & myelo-	Nil (Safety & tolerability)	Distribution volume of 11C-PBR28 binding to translocator protein reduced

			peroxidase activity<br><br>Microglial PET imaging		Sparse blood samples limited analysis
	3-hydroxy-3-methylglutaryl-coenzyme A (HMG-CoA) inhibition	Lovastatin[12]<br>NCT03242499	F-dopa PET scan	No correlation specified	Percentage change in the striatal 18F-dopa uptake ratio deteriorated less in the lovastatin group
**Mitochondrial function**	Improved mitochondrial biogenesis	Pioglitazone[13,14]<br>NCT01280123	Leukocyte PGC-1α and target gene expression, plasma interleukin 6 (IL-6) and urine 8-hydroxy-deoxyguanosine (8OHdG)	Association of concentrations of biomarkers with change in total UPDRS score	Negative study<br>Baseline or changes from baseline in biomarker levels not associated with rate of progression, Pioglitazone did not alter biomarker levels

	Increased urate, (antioxidant)	Inosine[15] NCT02642393	Serum urate, striatal dopamine transporter binding	No correlation specified	Negative study<br>Sustained elevation in serum urate from treatment<br>No difference in dopamine transporter binding from treatment<br><br>Urate levels rose in serum and CSF of treated groups
		Inosine[16] NCT00833690<br><br>*Serum urate below population median concentration (<5.8 mg/dL) used for inclusion	Serum & CSF urate	Nil (Safety & tolerability)	

	Improved mitochondrial function	Ursodeoxycholic acid[17] NCT03840005	ATP levels using ^{31}P MR spectroscopy	No correlation specified	Increase in free energy and inorganic phosphate levels in the treatment group
**Iron**	Iron chelation	Deferiprone[18] NCT02655315	Iron levels in substantia nigra and striatum (MRI T2* sequence), changes in brain-structure volume (three-dimensional T1 sequence), serum ferritin levels, density of DaT quantified with SPECT DaT, & serum prolactin	No correlation specified	Clinical worsening in treatment group<br>Nigrostriatal iron content decreased more in the deferiprone group, No inverse correlation between brain-structure volumes and iron content, Iron content outside nigrostriatal pathway similar in trial groups, no difference DaT change, plasma ferritin decreased & plasma prolactin increased with deferiprone

**Insulin resistance**	GLP-1 receptor agonists (reduced insulin resistance, reduced inflammation and alpha-synuclein aggregation)	Exenatide[19,20] NCT01971242	Measures of insulin resistance (Akt, and mTOR signalling pathway) in serum neuronal derived exosomes, CSF drug levels, DATSCAN uptake	Association with UPDRS 3	Positive clinical outcomes<br>Therapeutic drug levels in CSF<br>Exenatide treatment augmented insulin resistance pathways<br>Improvements in UPDRS 3 associated with downstream insulin resistance marker levels<br>Trend to improvement of DATSCAN signal in exenatide group

Abbreviations α -syn /α -synuclein; alpha-synuclein, ATP; adenosine triphosphate, c-Abl; Abelson tyrosine kinase, CNS; central nervous system, CSF; cerebrospinal fluid, GCase; DAT-SPECT; Dopamine transporter single-photon emission computerized tomography imaging, DOPAC; 3,4-Dihydroxyphenylacetic acid Glucocerebrosidase, HVA; Homovanillic acid, LRRK-2; Leucine-rich repeat kinase 2, MDS-UPDRS; Movement Disorders Society-Unified Parkinson Disease Rating Scale, PD; Parkinson's disease, PGC-1α; Peroxisome proliferator-activated receptor-gamma coactivator.

1. Pagan FL, Hebron ML, Wilmarth B*, et al*. Nilotinib Effects on Safety, Tolerability, and Potential Biomarkers in Parkinson Disease: A Phase 2 Randomized Clinical Trial. *JAMA Neurol*. Mar 1 2020;77(3):309-317. doi:10.1001/jamaneurol.2019.4200
2. Simuni T, Fiske B, Merchant K*, et al*. Efficacy of Nilotinib in Patients With Moderately Advanced Parkinson Disease: A Randomized Clinical Trial. *JAMA Neurol*. Mar 1 2021;78(3):312-320. doi:10.1001/jamaneurol.2020.4725

3. Pagano G, Taylor KI, Anzures-Cabrera J*, et al*. Trial of Prasinezumab in Early-Stage Parkinson's Disease. *N Engl J Med*. Aug 4 2022;387(5):421-432. doi:10.1056/NEJMoa2202867

4. Lang AE, Siderowf AD, Macklin EA*, et al*. Trial of Cinpanemab in Early Parkinson's Disease. *N Engl J Med*. Aug 4 2022;387(5):408-420. doi:10.1056/NEJMoa2203395

5. Jennings D, Huntwork-Rodriguez S, Henry AG*, et al*. Preclinical and clinical evaluation of the LRRK2 inhibitor DNL201 for Parkinson's disease. *Sci Transl Med*. Jun 8 2022;14(648):eabj2658. doi:10.1126/scitranslmed.abj2658

6. Jennings D, Huntwork-Rodriguez S, Vissers M*, et al*. LRRK2 Inhibition by BIIB122 in Healthy Participants and Patients with Parkinson's Disease. *Mov Disord*. Mar 2023;38(3):386-398. doi:10.1002/mds.29297

7. Mullin S, Smith L, Lee K*, et al*. Ambroxol for the Treatment of Patients With Parkinson Disease With and Without Glucocerebrosidase Gene Mutations: A Nonrandomized, Noncontrolled Trial. *JAMA Neurol*. Apr 1 2020;77(4):427-434. doi:10.1001/jamaneurol.2019.4611

8. den Heijer JM, Kruithof AC, van Amerongen G*, et al*. A randomized single and multiple ascending dose study in healthy volunteers of LTI-291, a centrally penetrant glucocerebrosidase activator. *Br J Clin Pharmacol*. Sep 2021;87(9):3561-3573. doi:10.1111/bcp.14772

9. Peterschmitt MJ, Saiki H, Hatano T*, et al*. Safety, Pharmacokinetics, and Pharmacodynamics of Oral Venglustat in Patients with Parkinson's Disease and a GBA Mutation: Results from Part 1 of the Randomized, Double-Blinded, Placebo-Controlled MOVES-PD Trial. *J Parkinsons Dis*. 2022;12(2):557-570. doi:10.3233/JPD-212714

10. Olson KE, Namminga KL, Lu Y*, et al*. Safety, tolerability, and immune-biomarker profiling for year-long sargramostim treatment of Parkinson's disease. *EBioMedicine*. May 2021;67:103380. doi:10.1016/j.ebiom.2021.103380

11. Jucaite A, Svenningsson P, Rinne JO*, et al*. Effect of the myeloperoxidase inhibitor AZD3241 on microglia: a PET study in Parkinson's disease. *Brain*. Sep 2015;138(Pt 9):2687-700. doi:10.1093/brain/awv184

12. Lin CH, Chang CH, Tai CH*, et al*. A Double-Blind, Randomized, Controlled Trial of Lovastatin in Early-Stage Parkinson's Disease. *Mov Disord*. May 2021;36(5):1229-1237. doi:10.1002/mds.28474

13. Investigators NETiPDF-Z. Pioglitazone in early Parkinson's disease: a phase 2, multicentre, double-blind, randomised trial. *Lancet Neurol*. Aug 2015;14(8):795-803. doi:10.1016/S1474-4422(15)00144-1

14. Simon DK, Simuni T, Elm J*, et al*. Peripheral Biomarkers of Parkinson's Disease Progression and Pioglitazone Effects. *J Parkinsons Dis*. 2015;5(4):731-6. doi:10.3233/JPD-150666

15. Parkinson Study Group S-PDI, Schwarzschild MA, Ascherio A*, et al*. Effect of Urate-Elevating Inosine on Early Parkinson Disease Progression: The SURE-PD3 Randomized Clinical Trial. *JAMA*. Sep 14 2021;326(10):926-939. doi:10.1001/jama.2021.10207

16. Parkinson Study Group S-PDI, Schwarzschild MA, Ascherio A*, et al*. Inosine to increase serum and cerebrospinal fluid urate in Parkinson disease: a randomized clinical trial. *JAMA Neurol*. Feb 2014;71(2):141-50. doi:10.1001/jamaneurol.2013.5528

17. Payne T, Appleby M, Buckley E*, et al*. A Double-Blind, Randomized, Placebo-Controlled Trial of Ursodeoxycholic Acid (UDCA) in Parkinson's Disease. *Mov Disord*. May 29 2023;doi:10.1002/mds.29450
18. Devos D, Labreuche J, Rascol O*, et al*. Trial of Deferiprone in Parkinson's Disease. *N Engl J Med*. Dec 1 2022;387(22):2045-2055. doi:10.1056/NEJMoa2209254
19. Athauda D, Maclagan K, Skene SS*, et al*. Exenatide once weekly versus placebo in Parkinson's disease: a randomised, double-blind, placebo-controlled trial. *Lancet*. Oct 7 2017;390(10103):1664-1675. doi:10.1016/S0140-6736(17)31585-4
20. Athauda D, Gulyani S, Karnati HK*, et al*. Utility of Neuronal-Derived Exosomes to Examine Molecular Mechanisms That Affect Motor Function in Patients With Parkinson Disease: A Secondary Analysis of the Exenatide-PD Trial. *JAMA Neurol*. Apr 1 2019;76(4):420-429. doi:10.1001/jamaneurol.2018.4304
